# Blunt Renal Trauma in a Pre-Existing Renal Lesion

**DOI:** 10.1100/tsw.2006.364

**Published:** 2006-02-28

**Authors:** G.V. Soundra Pandyan, Idris Omo-Adua, Mohammed Al Rashid, Ahmed Bakheet Zaharan

**Affiliations:** Department of Urology, Assir Central Hospital, ABHA, Saudi Arabia

**Keywords:** blunt renal injury, pre-existing renal lesion, renal cyst, stenting

## Abstract

A 70-year-old male presented with direct trauma to his loin with gross hematuria, as an isolated case of blunt renal trauma (BRT) due to a traffic accident. A pre-existing renal lesion (PERL) was strongly suspected by his past history of gross macroscopic hematuria and monotrauma to the kidney without other associated injuries. Spiral CT scan with contrast and a retrograde pyelogram (RGP) confirmed an occult complex renal cyst. The gold standard of CT diagnosis in this situation is stressed. Computed tomography is particularly useful in evaluating traumatic injuries to kidneys with pre-existing abnormalities. The decision on the initial course of conservative management, ureteral retrograde stenting to drain extravasation, and its final outcome are discussed. Radical nephroureterectomy was carried out by a transperitoneal approach with an early vascular control of the renal pedicle. A brief review of recent literature has been undertaken.

## INTRODUCTION

Pre-existing renal lesions (PERLs) associated with traumatic injuries increase the risk of blunt renal trauma (BRT) and these abnormal kidneys are easily injured, even with relatively trivial trauma. Symptoms are out of proportion to the nature of the injury in these cases. When a minor trauma results in renal injury, one should suspect an occult PERL. They may complicate an otherwise negligible renal trauma that may play an important role in the patient’s final outcome. In this case, we highlight an unusual presentation of an occult renal cyst ruptured by BRT. Preoperative suspicion of renal cell carcinoma (RCC) by the patient’s history and its confirmation by computed tomography (CT), and the initial dilemmas faced in early decision on conservative and surgical management with the final outcome are discussed.

## CASE HISTORY

A 70-year-old male Saudi presented to ER with a history of frank hematuria and right loin pain following a pedestrian automobile accident. He was hit by a car from behind with direct impact to his loin. He had past attacks of painless total hematuria 2 weeks prior, which had lasted for 3 days. On examination, his general condition was satisfactory with no pallor, pulse 90/min, and BP 110/70 mmHg. There were superficial lacerations over the scalp and cheek. Abdominal examination revealed a tender ill-defined mass in the right hypochondrium with right loin fullness. There was no urethral bleeding or associated boney injuries. Other systems were normal on examination. A Foleys catheter inserted drained 250 cc of bloody urine. Routine investigations showed the WBC: 11.5, Hb: 13.9 G/dl, ESR: 40 mm/h with normal renal functions, and an “O” negative blood group. Gross hematuria warranted further imaging procedures. Ultrasound of the abdomen showed a lacerated right kidney with a huge perinephric hematoma with poorly defined renal outline. A Grade 4 laceration of the right kidney with intrarenal and perirenal fluid collection, poor contrast enhancement (only a rim of anterior and superior part of kidney) was reported by helical CT (Fig. 1). Heterogeneous hypodense areas in the middle and lower pole of the right kidney gave a strong suspicion of a pre-existing renal mass (Fig. 2). As the patient was hemodynamically stable, we opted for initial conservative management with drainage of the collecting system. Cystoscopy and retrograde pyelogram (RGP) showed extensive extravasation of contrast from the collecting system with an ill-defined outline of the middle and lower pole of the right kidney (Fig. 3). In this case of Grade 4 renal trauma with a high suspicion of a possible malignant lesion and a stable patient, immediate surgical intervention was deferred at this juncture pending further re-evaluation. An 8F/28 JJ stent was inserted and the patient managed further with antibiotics, analgesics, IV fluids, and blood transfusion. After further interrogation and 48 h later, we received access to his old USG (Ultrasonography) film done 2 weeks prior, which showed a complex renal cyst, highly suggestive of RCC. Urine cytology was negative for malignant cells. We were considering FNAB (Fine Needle Aspiration Biopsy) to make a tissue diagnosis. During this 10-day period, the patient was doing well, but on the 11th day, he suddenly developed distension of abdomen, ileus, and sudden increase in the size of the mass. There was no response to conservative treatment by NGT (Naso Gastric Tube) aspiration and IV fluids; on the contrary, there was further increase in the size of the renal mass, with hypotension and fall of Hb. (8.6 gm) during the next 48 h. There was no significant response to blood transfusion. Repeat KUB (Kidney Urinary Bladder) x-ray showed gross shift of the JJ medially with increased renal area soft-tissue shadow (Fig. 4 - b). USG showed an increase in the size of the renal mass associated with fluid in the peritoneal cavity. Decision for emergency exploration was taken. Laparotomy through a mid-line incision revealed the liver, spleen, and rest of abdominal viscera to be normal. Early renal vascular control as described by McAninch[13] was achieved, followed by mobilization of the whole renal mass along with the Gerota’s fascia. Right radical nephroureterectomy was done. After opening the Gerota’s fascia, the specimen showed an extensively lacerated kidney with a large ruptured cyst involving most of the kidney (Fig. 5). Histopathology confirmed it to be a simple cyst with no evidence of malignancy. The patient had a stormy postoperative period. He developed hypoalbuminemia, anemia, chest infection, and ascities. These were managed by blood transfusion, 25% albumin infusions, hematopoietics, and specific antibiotics. He recovered well and was discharged on the 18th post-op day. He is doing fine 2 years to date.

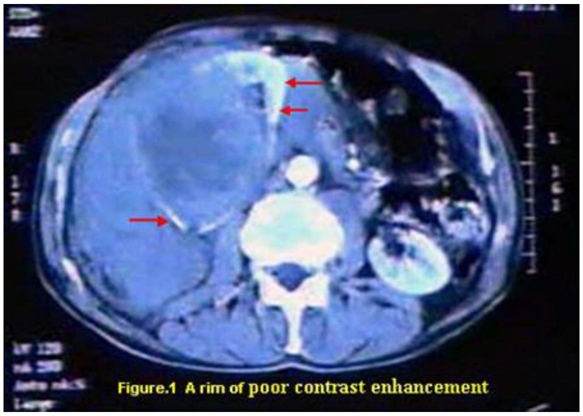

**FIGURE 1.**

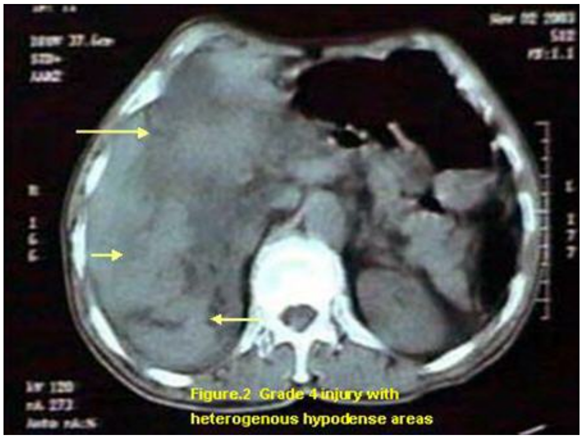

**FIGURE 2.**

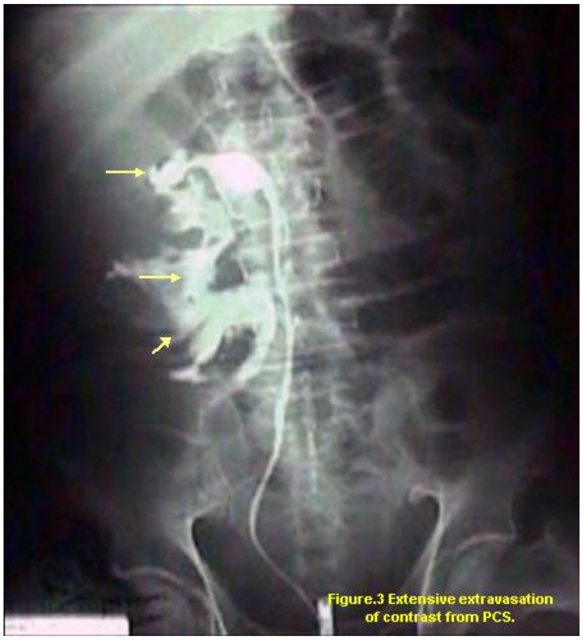

**FIGURE 3.**

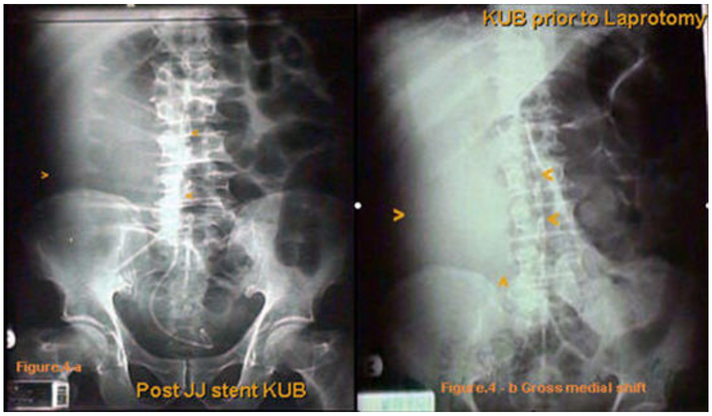

**FIGURE 4a and b.**

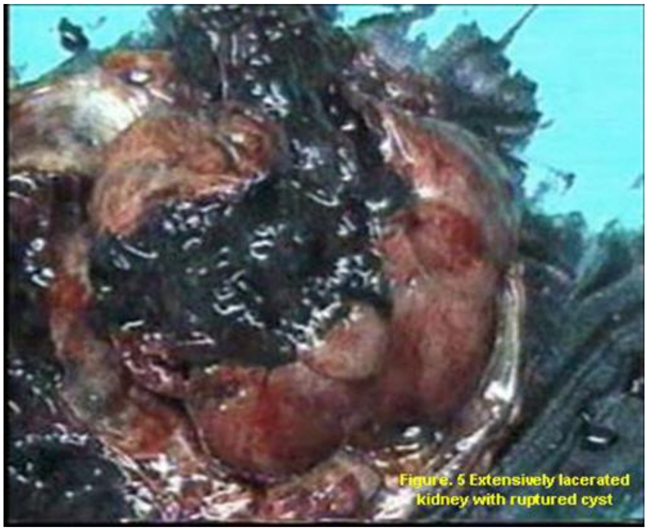

**FIGURE 5.**

## DISCUSSION

PERLs, although unusual, are known to present primarily as renal trauma. The incidence of their association with BRT is reported to be 4.4–19% in adults[1,2] and as high as 12.6–35% in children[1,3,4]. Even trivial trauma can result in serious renal injury. Trauma in PERL produces more symptoms than expected for the extent of the injury. Common PERLs are hydronephrosis (Pelvic ureteric junction obstruction, stone, and reflux), cysts, tumors, and abnormal kidney position. PERLs increase the vulnerability of kidneys in BRT. This predisposition has been attributed to decreased tissue strength in hydronephrotic kidneys and an altered tissue deformation of the renal cortex in the presence of a fluid-filled lesion (cyst, hydronephrosis). Increased susceptibility in the pediatric population can be explained by the relatively larger body proportion of pediatric kidneys and their lesser protection from the more pliable thoracic cage, weaker abdominal musculature, and less perirenal fat[2,5]. In BRT, the decision to image should be based on history, mechanism of injury, physical findings, laboratory studies, and clinical status. The best imaging modality at present in a hemodynamically stable renal trauma is spiral CT. It can identify parenchymal lacerations, urinary extravasation, perirenal hematoma, and vascular injuries. In addition, it helps to stage the injury, identify associated solid-organ injuries, pre-existing pathologies of the injured kidney, and also to document the function of the opposite kidney[1,6]. The present-day protocol in use is helical CT of abdomen and pelvis, with an arterial and/or portal venous phase from diaphragm to ischial tuberosities, and 10 min delayed images, kidneys through to ischial tuberosities[1]. CT is more sensitive and specific than IVP (Intra Venous Pyelogram), ultrasonography, or angiography. Qin et al. found that the positive rate during evaluation of 298 patients was 95.6% by CT, 90.9% by double-dose intravenous IVP, and 78.8% by ultrasound[8].

The management of patients with BRT has become increasingly conservative and surgical intervention is uncommon. The overall exploration rate for blunt trauma is less than 10%[9]. Primary conservative management is associated with a lower rate of nephrectomy, without any increase in the immediate or long-term morbidity[2]. The failure of conservative therapy is relatively low (5%)[10]. The management of abnormal kidneys unmasked by trauma is, to a large extent, dependent on the type of pathology. In a stable patient, the trend should be towards conservative treatment initially, even though most of the times surgery will be needed electively at a later stage. Giannopoulos et al. described 24 cases of PERL with BRT of which 3 were traumatic rupture of large cortical cysts; in two of the cases, surgical intervention was needed[7]. PERLs were found in 23/120 BRT cases by Schmidlin et al. Of those, 57% had to be operated on because of the underlying renal pathology[2]. Persistent urinary extravasation from an otherwise viable kidney after blunt trauma often responds to stent placement and/or percutaneous drainage. Ureteral stents may avoid the need for surgical exploration in patients with Grade 4 renal trauma who develop recurrent gross hematuria, flank pain, and persistent extravasation secondary to clot obstruction[11]. Repeat imaging 2–4 days after trauma minimizes the risk of missed complications, especially in grades 3–5 blunt renal injuries[12]. Definitive surgical management in PERL depends on the underlying pathology.
